# Fracture of the Patella from Muscular Action*I am just now preparing a paper on “Fracture of the Patella,” based upon an analysis of probably 150 cases. While referring to my notes I found the case reported here, which has some points of practical interest, and which seem worthy of publication entire. F. H. H.

**Published:** 1879-09

**Authors:** Frank H. Hamilton

**Affiliations:** New York


### FRACTURE OF THE PATELLA FROM MUSCULAR ACTION.*

GOOD FIBROUS UNION—REFRACTURE FROM MUSCULAR ACTION—IMPERFECT FIBROUS UNION, WITH SEPARATION OF 4½ INCHES—NO SUBSEQUENT IMPROVEMENT, OWING TO ATROPHY OF THE MUSCLES OF THE THIGH, UNTIL ALL KNEE SUPPORTS WERE LAID ASIDE.

REPORTED BY FRANK H. HAMILTON, M. D., NEW YORK.

AFTER Surgeon Myers consulted me he sent to me at my request, the following account of his case, from the records of the hospital at Yokohama, Japan, with corrections made by himself:

T. D. Myers, Assistant Surgeon U. S. N., aged twenty-eight years. Fracture of right patella. "Assistant Surgeon T. D. Myers, returning from the U. S. S. *Kearsarge*. . . . In attempting to get out of the steam launch, when quite a heavy sea was running, was thrown on to the platform of the gangway ladder so that his whole weight was received on his right leg in a semi-flexed position, and in that manner the patella was fractured by direct muscular action." (Copy from hospital ticket.)

Fracture occurred on the evening of the 19th of May, 1874, in the harbor of Yokohama, Japan.

Patient was placed in bed (on board the U. S. Flagship *Hartford*,) with the whole limb elevated, and the fragments approximated by a figure of eight bandage.

On May 21st patient was removed to the U. S. Navy Hospital, on shore, in the city of Yokohama, Japan.

Notes in quotation marks are copied from the "Hospital Journal."

"On entrance into Hospital the right patella, which is the bone fractured, was approximated by means of a figure of eight bandage, while a posterior splint of wood, with two side splints of pasteboard were applied to steady the limb."

* I am just now preparing a paper on "Fracture of the Patella," based upon an analysis of probably 150 cases. While referring to my notes I found the case reported here, which has some points of practical interest, and which seem worthy of publication entire.

F. H. H.

"May 24th.—Removed all bandages, re-applying them as above, adding a longer posterior splint. Patient is placed on a fracture bed and his limb is constantly kept on a single inclined plane. General health perfect."

"May 27th.—Applied Lausdale's apparatus."

"May 31st.—Doing well. Obliged to take morph. sulph. grs. ss. ($\frac{1}{2}$) at night to relieve pain caused by the apparatus. Also applied roller to whole limb to quiet muscular action."

"June 3d.—Doing well. The patella presents some signs of union."

"June 7th.—Doing well."

"June 10th.—Removed two days since Lausdale's apparatus, retaining only posterior splint, re-applying bandage as at first."

On June 8th Lausdale's apparatus was removed because the integument under the points of pressure, commenced to ulcerate. The pain was so severe that, although the patient was taking a half grain of the sulphate of morphia each night, he was unable to sleep. Patient did well from this time. Reparative action progressing uninterruptedly.

"June 21st.—Making passive motion. Removed the wooden posterior splint and applied one of felt, moulded to the shape of the limb."

"June 28th.—Continue the use of passive motion, and the application of the posterior felt splint. The bone has united by ligament, being separated something over half an inch on the inner side and about one-fourth of an inch on the outer. Walks about on crutches at present."

The ligament was shorter than stated above, and was V shaped or triangular in form, the base of the triangle being outwards.

"July 26th.—Continues to improve, can bend the limb at right angles, when the uniting ligament is found to be quite firm and strong. Continue the use of elastic knee cap and posterior splint with bandage."

The elastic knee cap mentioned in above note was applied some five days before, *i. e.*, about the 21st of June.

To obtain the extent of motion noted above, great care was necessary, and the limb was slowly and carefully flexed. The fracture, which was transverse, and situated so low down, *i. e.*, near apex of the bone, that the fractured surfaces were very small, and the ligament, of course, corresponded with these surfaces.

"July 29th.—Removed posterior splint and bandage."

Patient walked well without any support other than that given by an ordinary elastic knee cap.

"Aug. 3d.—Returning to Hospital last evening at 10 P. M. patient slipped, and falling produced a partial rupture of the uniting ligament between the fragments of the broken patella. The knee became immediately greatly swollen. Applied a roller bandage."

The above note is not entirely accurate, in this, the fall followed the rupture of the ligament, *i. e.*, in the effort to save himself when slipping the rupture took place.

"Aug. 5th.—There is considerable echymosis about seat of injury. Removed roller and applied starch bandage."

"Aug. 7th.—The swelling of the knee subsiding, starch bandage has become so loose as to necessitate its removal, which was done. The ends of the patella were then approximated as closely as possible by means of adhesive plasters above and below, and a dry roller applied, over which was placed plaster of Paris bandages."

"Aug. 8th.—The dressing has been strengthened by a posterior felt splint and additional plaster of Paris bandages."

"Aug. 14th.—Removed plaster of Paris splint and found the fragments in good apposition. Re-applied bandage as before.

"Aug. 19th.—Again removed the dressing, together with adhesive plasters. The fragments were within one-third of an inch of each other by measurement. Applied dressing as before, together with adhesive strips."

"Aug. 29th.—Fragments one and one-third inches from each other. Plaster Paris dressing."

"Aug. 31st.—Renewed dressing, fragments quite widely separated, and it is difficult to detect any union. Removed plaster Paris dressing. Applied adhesive straps and a posterior splint of sole leather, after making passive motion."

"Sept. 6th.—Removed adhesive plaster. No new developments since last notation."

"Sept. 13th.—Removing adhesive plaster every third day, making at such times passive motion."

"Nov. 1st.—Employing adhesive plaster and elastic stocking and knee lap, together with posterior splint of sole leather. The fragments are widely separated."

On the 23d of November, patient left Yokohama, Japan, for the United States. Condition unchanged as noted above. Fragments about two inches from each other, with little or no union between them. While passive motion was being made, as mentioned above, the upper fragment was steadily pressed down to prevent, if possible, any tension on the connecting fibres, if any existed. Patient arrived in the United States, after a journey of over two months, by steamship, without any further change than a slight increase of the distance of separation of the fragments. General health somewhat impaired by long confinement and loss of exercise.

When Surgeon Myers consulted me, March 17, 1875, I advised removal of the knee supports, careful use of the limb in walking, passive motion, frictions and electricity, with the view of developing the muscles of the thigh; my experience in other similar cases having been, that the remaining attachments of the quadriceps to the sides and fronts of the tibia and fibula, through the aponeurotic expansions of this muscle, would in a great measure restore the power of extension and of locomotion. I did not then discover any fibrous bond between the fragments.

The following letter from Surgeon Myers will give the results of this experiment:

1136 GIRARD STREET,

PHILADELPHIA, PA., May 23d, 1875.

*Sir* :—Since consulting you on the 17th of March, 1875, I have steadily pursued the plan of treatment suggested by you on that occasion. On March 23d, I took off the posterior splint, and have not re-applied it since. On the morning of its removal I forcibly flexed the knee by accident, and feared that I had severed the few remaining fibres of tendon; but after the inflammation subsided, I found that the fragments were not any more widely separated than before. And I now believe, that the only effect of the flexion was to break up some slight adhesions about the insertions of the inner ham string muscles. This accident confined me to my bed for three or four days.

The function of the limb has gradually returned, till now I am able to walk very well with very little, or, no limping. The limb is weak and soon becomes tired. In the past two months the fragments have separated more than an inch farther than when you saw them; there is now full four inches between the pieces. (According to my measurement it was 4½ when I saw him.—F. H. H.) The Atrophy of the extensor muscles is gradually disappearing. The increased tonicity of these muscles, with the consequent greater tension on the connecting fibres will account for the further separation of the fragments.

I have been applying electricity daily as directed.

I have taken the liberty to mail to your address notes from the "Hospital Journal" with corrections, in my case. Hoping, that they may prove sufficient for any purpose of your own, I remain with much gratitude,

Yours respectfully,

T. D. MYERS,
Asst. Surgeon U. S. Navy.

Prof. FRANK H. HAMILTON.

